# How Low-Income Mothers Select and Adapt Recipes and Implications for Promoting Healthy Recipes Online

**DOI:** 10.3390/nu11020339

**Published:** 2019-02-05

**Authors:** Lauren N. Tobey, Christine Mouzong, Joyce Senior Angulo, Sally Bowman, Melinda M. Manore

**Affiliations:** 1 Extension Family and Community Health, College of Public Health and Human Sciences, Oregon State University, Corvallis, OR 97331, USA; christine.mouzong@oregonstate.edu (C.M.); bowmans@oregonstate.edu (S.B.); 2 Extension Family and Community Health, College of Public Health and Human Sciences, Oregon State University, Astoria, OR 97103, USA; joyce.senior@oregonstate.edu; 3 School of Biological and Population Health Sciences, College of Public Health and Human Sciences, Oregon State University, Corvallis, OR 97331, USA; melinda.manore@oregonstate.edu

**Keywords:** low-income mothers, focus group, nutrition, Supplemental Nutrition Assistance Program (SNAP), social media, recipe, social marketing, children, feeding behavior, website development

## Abstract

We describe a 5-year (2011–2015) qualitative evaluation to refine the content/delivery of the Food Hero social marketing campaign recipes to low-income mothers. Objectives were to: (1) identify characteristics looked for in recipes; (2) determine recipe sources; (3) understand motivation for seeking new recipes and recipe adaptations; and (4) identify recipe website characteristics users valued. Nine focus groups (*n* = 55) were conducted in Portland, Oregon. Participants (35–52 years) were primary caregivers for ≥ one child, the primary household food shoppers/preparers, enrolled in the Supplemental Nutrition Assistance Program (SNAP) and able to speak/read English. Participants reported having “go-to” family recipes and regularly searching online for new recipes, especially those using ingredients available/preferred by family members. Recipe websites with highest appeal were polished and engaging to mothers/children, offered user-ratings/comments and were reachable from search engines. Results identified key recommendations: (1) understand the target audience; (2) aim to add healthy/customizable recipes to family “go-to’ recipe rotations and understand the impact of generational influences (e.g. how mothers/grandmothers cooked) on family meals; and (3) create websites that meet target audience criteria. Seeking the target audience’s input about the content/delivery of recipes is an important formative step for obesity-prevention projects that include healthy recipes.

## 1. Introduction

Currently, 39.8% of United States (US) adults are obese (body mass index [BMI] ≥30 kg/m^2^), while 31.8% are considered overweight (BMI = 25 to <30 kg/m^2^) [1]. One factor contributing to these high obesity/overweight rates may be the changing US eating behaviors. Data from the US Department of Agriculture show that between the years 2004–2017, in constant dollars, annual expenditures on food away from home (FAFH) surpassed expenditures on food eaten at home. The data also show that since 2010 the gap is expanding annually [2]. Research shows a positive association between eating FAFH and increased obesity and overweight, whereas home-prepared meals (HPM) are associated with lower intakes of energy (kcal), sugars and saturated fat and a reduced risk for type 2 diabetes [3,4,5]. Lower body weight and body fat levels have also been linked to HPM intake [6]. Mills et al. found that adults in the United Kingdom (*n* = 11,396), who frequently consume HPM (> 5 times/week), were 28% less likely to be overweight and 24% less likely to have excess percent body fat compared to those who consume HPM infrequently (<3 times/week). HPM also contributed to a more nutrient dense diet [6,7]. Tiwari et al. surveyed US adults (21 to 55 years), who were the primary food shoppers in their household (*n* = 437) and found frequent HPM to be associated with increased compliance to the *2010 Dietary Guidelines for Americans* without significantly increased food expenditures [7]. Conversely, frequent consumption of FAFH was associated with both lower DGA compliance and higher food expenditures [7]. Thus, promotion of healthy HPM can improve overall dietary intake and may be an important component of obesity-prevention and weight-management programs.

Despite an increasing trend toward eating out, many US adults, especially women, continue to cook at home. Research by Taille, using nationally representative American Time Use Study data of US adults (18–65 years; *n* = 139,219) in 2003 and 2016, reported that 67–70% of women cooked at home for 47–50 min/day and 35–46% of men cook for 15–20 min/day [8]. Additionally, women with lower levels of education were more likely to cook and spend more time cooking per day compared to women with higher levels of education—Taille used educational attainment as a proxy for income, per data limitations. For example, in 2016, women with less than a high school diploma reported cooking for 64 min/day, whereas women with a college degree reported cooking for 43 min/day. Adults with one or more children <18 years at home were also more likely to cook and for more minutes per day; both data points increasing from 2013 to 2016 (in 2016, 68% cooked at home versus 53% for those without a child/children at home and for 49 versus 28 min/person/day) [8].

One strategy nutrition educators use to promote preparation of healthy HPM is to share healthy recipes with their targeted audiences using a variety of methods (e.g., curricula, social marketing campaigns, social media, websites, food tastings and cooking demonstrations) [9,10,11]. Formative research shows adults are interested in receiving recipes to support healthy eating in a variety of formats, including online and are currently accessing recipes online and offline to inspire their HPM [12,13]. For example, Doub et al. conducted an online survey of adult mobile device users (*n* = 583), who were ≥50% responsible for household grocery shopping and meal planning and found that many participants used multiple sources for meal ideas (e.g. family and friends, cookbooks, Internet) [13]. They found the use of cookbooks as a resource for meal ideas was positively associated with HPM frequency, compared to participants who did not use cookbooks [13]. Similar trends for Pinterest and food company websites were found, though they did not achieve significance [13]. However, research examining recipes to determine if they meet national nutritional guidelines (e.g., the DGAs) is still in its infancy [13]. Initial research assessing the nutritional quality of recipes found on popular online food sites has focused on blogs. This research has found that most recipes are nutritionally limited and few feature a fruit or vegetable (FV) as the main ingredient [14,15]. Although healthy recipes are used to encourage HPM and healthier eating, there is no consensus regarding best practices for types of recipes to offer or how to most effectively deliver them online to the target audience.

In 2009, in response to the need to reduce obesity and increase FV intake in Oregon families, the Oregon State University (OSU) Extension Service developed, piloted and launched the Food Hero social marketing (SM) campaign, which has now been ongoing for nearly ten years [12]. The Food Hero target audience is SNAP-eligible mothers and other members of the communities in which they reside. A central goal of Food Hero is to offer fast, tasty, healthy and low-cost recipes that appeal to mothers and their children and include a variety of FV. Food Hero recipes are delivered in a variety of ways, including by nutrition educators (*n* = 105) directly to >268,000 Oregonians per year at events throughout Oregon and online to >1.8 million users/year in 193 countries, including all US states.

The Food Hero SM campaign is guided by social cognitive and diffusion of innovation theories, the social-ecological model of behavior change and the eight SM benchmark criteria (i.e., behavior, customer orientation, theory, insight, exchange, competition, segmentation and methods mix) [16,17,18]. SM is “a process that applies marketing principles and techniques to create, communicate and deliver value in order to influence target audience behaviors that benefit society (public health, safety, the environment and communities) and the target audience” [19]. The SM benchmark criteria inform ongoing Food Hero evaluation/development efforts, as SM has been found to be more effective at changing eating behaviors and preventing obesity in youth when at least five of the benchmark criteria are used [20,21]. The SM benchmark criteria support continual assessment of the target audience to adjust campaign content to address changing needs, including classifying the target audience into “segments” with common characteristics and targeting campaign efforts accordingly, thereby avoiding a “one size fits all” approach [16].

To our knowledge there are no published studies focused on describing what characteristics low-income mothers look for in recipes, including characteristics valued by mothers when selecting recipes online. This paper describes a 5-year (2011–2015) qualitative Food Hero SM campaign evaluation aimed at refining the content and delivery of healthy recipes for low-income families. A qualitative approach was used to elicit a descriptive and personal narrative from a sample of SNAP-enrolled mothers living in Oregon. This study had four key objectives: (1) identify characteristics mothers looked for in recipes; (2) determine recipe sources mothers used; (3) understand mother’s motivation for seeking new recipes and how recipes are adapted; and (4) identify the recipe characteristics valued by mothers when selecting recipes online. 

## 2. Materials and Methods 

### 2.1. Study Design

Nine focus groups (FGs) with SNAP-enrolled mothers/caregivers were conducted from 2011 to 2015 (Table 1) at professional focus group facilities in Portland, Oregon. A private research firm (Davis, Hibbits, & Midghall, Inc., Portland, Oregon) managed screening of participants, moderated FG discussions, transcribed response data and used an iterative approach to prepare a summary of key findings of each session. OSU researchers managed recruitment of participants, created the FG interview guides, observed FGs to ensure accuracy of transcriptions and conducted coding and data analysis of response data. 

### 2.2. Recruitment

Eligible participants were recruited from the tri-county area of Portland, Oregon (where 34% of Oregonians on SNAP reside) through flyers posted and distributed in heavily used SNAP offices in Washington and Multnomah Counties. Using purposive sampling techniques, flyers were also emailed to a subset of SNAP-eligible participants who consented to future contact after responding to previous Food Hero questionnaires [22,23]. Interested participants called the telephone number provided and were screened for eligibility parameters: mothers or female guardians ≥18 years, residing with at least one child ≤18 years; enrolled in Oregon SNAP; primary food shoppers/preparers in their households; could speak and read English and had not previously participated in a Food Hero FG session. Prior to the event, participants received a mailed information packet. After the FGs each participant received $25.00 for their participation. FGs were conducted during the school months to help normalize the findings of participants’ reported habits during a typical school week.

### 2.3. Instruments and Procedures

Each FG was asked 18–25 scripted questions and follow-up probes to examine: (1) home prepared meals self-efficacy, frequency and food preparation attitudes; (2) healthy eating definitions and perceived barriers; (3) recipe use and selection; (4) current use of recipe websites and desired traits in website content and style; and (5) Food Hero campaign elements, messages and delivery mechanisms. Social cognitive theory constructs, such as perceived self-efficacy—the belief in one’s ability to organize and perform the actions required to attain a desired outcome—, were used to inform the interview scripts (see Supplementary Materials) [24,25,26]. Each of the five sets of FGs (FGs A-E, see Table 1) was initiated to test new and emerging Food Hero campaign elements and messaging over a 5-year period as the budget allowed. The OSU Institutional Review Board approved and reviewed study protocols and question guides for each set. The protocol stated that each FG session last ~90 min, be held at a professional FG facility and used a trained moderator. All FGs were audiotaped and transcribed verbatim and all participants verbally consented to taping before each session began.

### 2.4. Data Analysis

OSU researchers independently reviewed the research firm’s summary reports and transcripts and met to compare initial observations. Using the social cognitive theory and SM benchmark criteria, researchers identified seven broad conceptual coding categories [23]. Data were analyzed using NVivo qualitative analysis software (version 11.2.0, QST International (Americas), Inc., Burlington, MA, USA) and themes were applied using a constant comparison method of line-by-line coding. As additional themes emerged during the coding process, researchers compared and reconciled coding differences before revising and finalizing the codebook. Thematic saturation was established once a theme or sub-theme occurred in at least four individual participant responses and was mentioned across three or more paired sets of FG sessions [27]. 

## 3. Results

The nine FGs (*n* = 55) had 4–9 participants each. Overall, the participants (35–52 years) were mothers (89%) who reported approximately two children of school age living at home (see Table 1). Only six of the primary caregivers were aunts or grandmothers. Self-reported ethnicity data were available for 41 of 55 participants, which represented 75% of the sample: 54% white, 37% black, 5% Hispanic; 5% Asian.

Three salient themes emerged from the data: (1) home meal preparation habits and characteristics of “go-to” family recipes, including generational influences; (2) sources of and motivation for seeking new recipes and methods of adapting recipes; (3) desirable characteristics of recipe websites. Only sub-themes that achieved saturation criteria were categorized and reported in the results.

### 3.1. Theme 1: Home Meal Preparation Habits and Characteristics of “Go-To” Family Recipes 

When asked to describe their cooking habits, typical participants reported that they enjoyed cooking, that they made HPM daily, that the family rarely ate out because of budgetary constraints and that there was a family generational influence on their recipe choice and use. Participants indicated that deciding what to cook frequently depended on turning to reliable “go-to” family recipes. In order of preference, the most commonly mentioned “go-to” recipes included pasta/spaghetti, stir-fry, tacos/quesadillas, stews, meatloaf and hamburgers. Participants identified the following required criteria for their “go-to” family recipes:

- Inexpensive, Easy and Appealing. Recipes needed to include inexpensive ingredients that appealed to most or all family members and could be prepared with minimal time and effort and in some cases with or by children. Participants (~80%) seldom experimented with new recipes unless they could be prepared using familiar ingredients they were confident their families would eat. Many reported preparing “go-to” recipes from memory.
- Customizable and Kid-Friendly. Participants often mentioned family members’ ingredient aversions, including aversions to specific foods (wild/brown rice, peas, mushrooms, onions, tomatoes), food groups (vegetables, meat) or textures (slimy). An example of a common sentiment was, *“I like to cook things that my daughter will enjoy and eat and she’s a picky eater.”* Participants identified their top strategy to minimize ingredient aversions as choosing recipes that allowed them to easily substitute ingredients they knew family members, especially children, would prefer. Participants also managed ingredient aversions by engaging children in meal planning and cooking. Recipes that combined both strategies were the most common “go-to” favorites across all groups. As one participant explained, *“I like to cook tacos because they’re easy… and all the kids will eat them. If one only wants meat and cheese, they can have meat and cheese. If one likes something else, you can throw everything out there and they can make them. Put out the taco bar and everyone makes their own.”*
- Healthy. Participants frequently stated that “go-to” recipes should be healthy because they wanted healthier eating for themselves and their families, including to comply with any medically prescribed dietary restriction/s of a family member. However, their definitions of healthy eating varied widely, for example, going dairy-free or gluten-free; using organic FV when possible; or occasionally substituting ground turkey for beef in a main dish. One participant captured the cohort’s commonly cited criteria in her definition of healthy eating: *“Whole grain, fiber… so, a variety of the different food groups. And organic if you can afford it. No sugar. Lots of fruits/veggies. Lots of water.”* Comparing participants “go-to” recipes to their definitions of healthy eating revealed several inconsistencies. For example, some participants voiced healthy eating aspirations, yet described “go-to” recipes that were nutritionally limited, such as: *“Baked potato soup. It’s so easy. You just make mashed potatoes and put cream of chicken and celery in there and broccoli. Then, you bake it and add sour cream—you put that on top. It doesn’t have meat, it’s one meal and there’s a vegetable inside it.” “Macaroni and cheese but like homemade, from scratch. I make big portions to last.” “I have two things that I am known for: my chocolate chip cookies and then my fried fish.”*
- Culturally Diverse. Throughout the FGs, participants named favorite dishes from many cultures, including Indian, Mexican, Chinese, Vietnamese, Italian, Russian, Southern US Creole, soul food, Japanese and Thai. For example: *“I like to know about some of the Chinese recipes and the Vietnamese and even any other culture, just how they came to be.”*

Although the FG guides did not direct participants to discuss family generational influences on their recipe choices, these influences on participants’ home meal preparation behaviors and recipe selection emerged repeatedly in responses. Participants were especially likely to bring up a mother’s or grandmother’s influence when describing how they decided what to cook for their families. One participant explained how her mother’s “go-to” meal repertoire had become folded into her own HPM decision-making process: “*I go back to what time availability I have and what do I have on hand and then I do a lot of creative [cooking]. Or, I go back to things that my mom made, which are a lot of creative-type recipes that you didn’t have to measure. You just kind of put it together. It wasn’t a recipe as far as measurements. You take a little bit of this and a little of that*.” Although those who spoke of this generational connection largely shared positive memories associated with learning to cook with family members, they disagreed about the importance of passing on family cooking traditions—including recipes—to their own children. About half of this group spoke of deliberately adopting healthier eating practices than those they were exposed to growing up: “*I thought [my weight] was just hereditary, because I have always been heavier. But it’s just that my family culture was so centered around food and celebration, so it was always something*.”

### 3.2. Theme 2: Sources and Motivation for New Recipe Use and Adaptation 

Participants identified their sources for new recipes, their reasons for seeking them out and the methods they used to adapt these recipes to fit their family’s needs. Each of these is described below.

**Figure 1:**
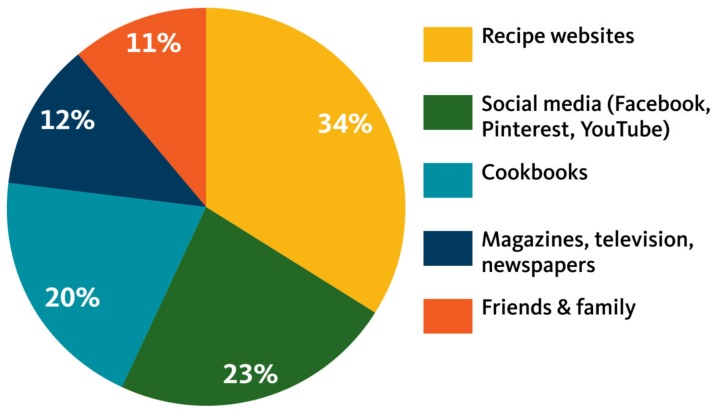
Sources for new recipes.

**Figure 2:**
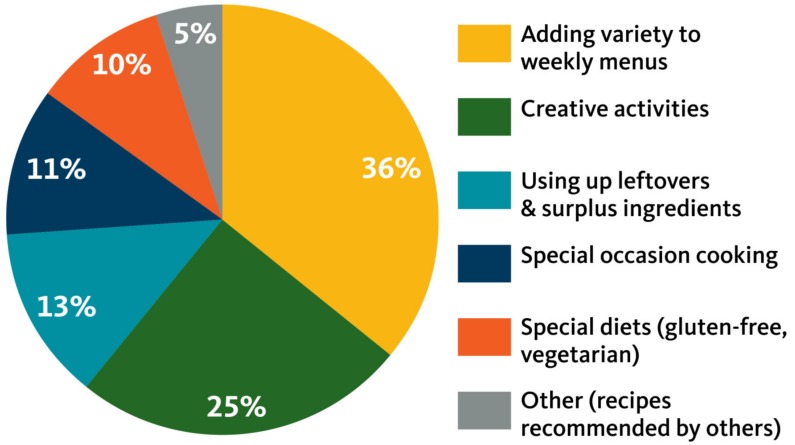
Motivation for finding recipes.

- Sources of New Recipes and Meal Ideas. Most participants (~90%) reported regularly using the Internet for meal ideas and recipe searches. In order of significance, the primary sources of new recipes were: websites, social media (especially Facebook, Pinterest and YouTube); cookbooks; magazines, newspapers and television channels such as the *Food Network*; and friends and family (Figure 1).
- Motivation for Seeking New Recipes. There were five main reasons participants sought new recipes: (1) to add variety to their meal routine while meeting family food preferences; (2) to engage in a creative activity involving food (e.g., baking cookies or learning how to make tamales); (3) to find new ways to use up surplus ingredients or leftovers (e.g., leftover chicken or garden vegetables); (4) to prepare foods for special occasions such as birthdays or holidays; and, (5) to prepare foods for a special diet such as gluten-free, vegetarian, low sugar or vegan (Figure 2). Discussing common reasons why families might seek out a new recipe for creative cooking, one participant said: *“Every so often [we’ll use a new recipe], like my daughter and one of her friends, just this weekend, wanted to make sugar cookies. They just looked it up online, just pulled it up and put in ‘sugar cookies.’”*
- Methods of Adapting Recipes. As previously described, participants typically reported adapting new recipes by substituting ingredients family members preferred, healthier ingredients, and/or ingredients they had available. In describing how she commonly adapted recipes, one mother commented, *“Just that I can put my own flavor and spin on it, like if I don’t stick to a recipe and kind of can change it up and do different things with it to suit the taste of the people that I’m feeding it to.”* Others echoed this sentiment across groups: *“There’s a lot of meat [in these recipes] but a lot of that I’d just change. I’d make it different. I’d probably use tofu. We use a lot of tofu.” “I have something in a recipe that I never heard of before and I couldn’t find it anywhere. So I just eliminated it and didn’t use it and it seemed fine. You can substitute concentrated apple juice [for] oil, which is a cheaper, healthier way to do a lot of things.”*

### 3.3. Theme 3: Desirable Characteristics of Recipe Websites

**Figure 3:**
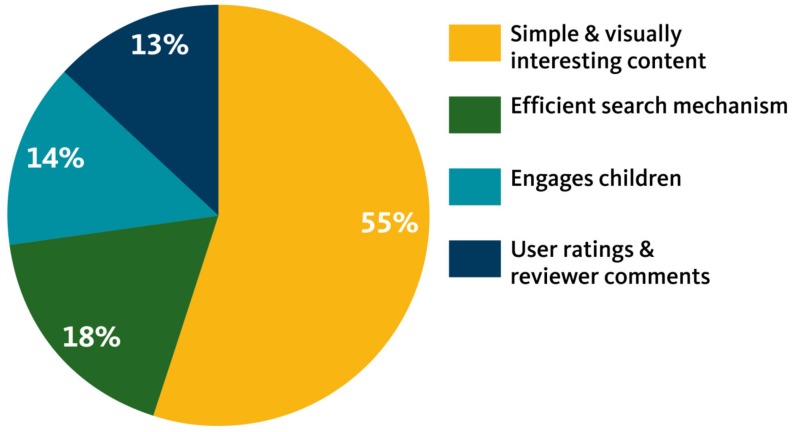
Characteristics desired in a recipe website.

For the majority of participants, the wide variety of options available on the Internet made online searching a highly salient method for finding recipes. When describing what they looked for in recipe websites, their responses fell into the four categories described below (Figure 3).

- Simple and Visually Interesting. This was the top characteristic of preferred recipe websites. Participants stressed the importance of balancing a simply formatted recipe website with visually interesting content: “*The whole process is telling you whatever you’re looking at, straight and to the point—click and you go to the next one, reading a little bit, no more than five lines—three, four or five lines.”* Bright, colorful, eye-catching pictures were also an important feature for participants and their children. One participant described a website she used often: *“When you get on it, it’s very bright and visual and you’re interested in looking at the pictures and seeing what they’re doing.”* In contrast, reasons not to revisit a website included overly complex recipe instructions and cooking tips or the perception they were not the intended audience. For example, one woman gave this evaluation of a popular website: “*Chef.com isn’t one of my favorites. It really was written for high-level home cooks, like the ones that have every kind of tool. Foo-foo. I think that just doesn’t work for a family.”*
- Efficient Search Mechanism. Participants generally searched for recipes from search engines like Google using a specific key word(s) or ingredient(s) versus by recipe name, yet both methods were used. As one participant elaborated, “*Say I have got a bushel of broccoli or something. I could click on broccoli and I’d see something with broccoli.”* Another explained, “*I like to garden and I have this big garden. It’s like, ‘okay, I’ve got all this squash. What am I going to do?’”* Being able to search by recipe name was also mentioned: “*If I do a 7-UP cake, I put in ‘7-UP’ and it will give you the different recipes. I’ll go through them and try to find the simplest.”* Some reported using multiple key words to increase the number of recipes in the search results: “*If you type in, ‘ways to make quinoa,’ then you have five or six websites that will pop up.”* Still others reported narrowing the search field by making the query more specific; *“I put ‘simple’ or ‘easy’ so they give you the most simple thing that comes up first.”*
- Engages Children. Participants preferred recipe websites with child-centered content and a design that allowed adults to use recipes side-by-side with their children. One mother, in explaining her idea of a child-centered website, said, *“A database that has the fruits and vegetables or that would match colors or have things that would be appropriate for kids as well as adults.”* However, participants reported that they did not typically find this characteristic in the recipe websites they viewed/used. Participants often stressed the importance of having content that could appeal to a range of children’s ages and abilities. As one participant explained, “*I have [a child] who is ready to cut the tomatoes herself and then one that has no idea what he’s doing, so something simpler that is for younger kids and then something as they get older in stages—kind of like in school.”* Child-friendly ideas mentioned across groups included the use of bright, eye-catching content and interactive animated characters to guide children step-by-step through recipe preparation.
- User Ratings and Reviewer Comments. Favorite recipe websites also included a user-rating system. Participants reported looking for a rating system—either star-ratings or text-based reviewer comments—before making a recipe. They relied on these systems to decide whether a recipe had high user approval or if users had given tips or provided ideas for substitutions to adapt or improve a recipe. For example, explaining why she liked using *Allrecipes.com*, a popular website among FG participants, one woman said: “*They’ve got the top comments. I looked up jerked chicken and they said, ‘Well, this recipe is good but all you need to do is just subtract this or this...’”* The majority of participants talked about reading online recipe comments but not choosing to post comments themselves: “*But I don’t want to post it, because I guess the constructive criticism, like if somebody was like, ‘Ugh, that looks nasty’…well, my son liked it, so it’s okay.” “I would probably read them but I wouldn’t post them.”*

## 4. Discussion

Findings from our FGs were used to refine the content and delivery of recipes through the Food Hero SM campaign, specifically to SNAP-enrolled mothers. Our research yielded three key recommendations for disseminating recipes to low-income audiences through a SM campaign: (1) understand the target audience to better define and support the recipe characteristics they may be looking for; (2) aim to add healthy, customizable recipes to family “go-to’ recipe rotations and understand generational influences on family meals; and (3) when delivering recipes online, create website content and design that meet the criteria the target audience seeks. 

When asked to describe cooking habits, participants reported enjoying cooking, making HPM daily and rarely eating out with their families because of budgetary constraints. Research consistently shows that US women of all backgrounds and income levels do the majority of HPM cooking; however, while most of the participants in our study reported cooking daily, the cooking frequency reported nationally among low-income US adults varies widely [8,28]. For example, Wolfson and Bleich, using nationally representative data from 2007–2010 (*n* = 9,569) found that low-income participants (*n* = 2,768) reported cooking either more frequently (24% cooked dinner 6–7 times/week) or less frequently (22% cooked dinner 0–1 times/week) as compared to those with higher incomes (*n* = 6,801), who cooked a moderate amount (86% cooked dinner 2–5 times/week) [5]. Our FG participants reported cooking frequently to save money but they also enjoyed cooking as a creative outlet and for the novelty of selecting meal ideas from a wide range of culturally diverse recipes. Previous research also reports that mothers choose HPM to save money and that HPM can be a lower-cost alternative to FAFH; however, some women are conflicted about the pressures that come with cooking and do not necessarily enjoy cooking or are lacking in food preparation skills or supplies [7,29,30,31,32]. Since mothers cook at different frequencies and view the role of cooking HPM differently, educators should first gauge their target audience’s current attitudes, barriers and self-efficacy toward achieving their ideal HPM. They should then aim to meet them at their current level of interest for and readiness to, adopt new recipes or else focus on a different component of HPM.

The majority of our FG participants reported consulting recipes for HPM. Participants all had an existing set of “go-to” recipes, many of which they cooked from memory but most also sought out new recipes and were confident about customizing both new and existing “go-to” recipes to their family’s liking. Our finding that mothers rely on “go-to” recipes is similar to Raskind et al., who interviewed (*n* = 40) women (mean 25 years) living in southwest Georgia regarding their food sources at the grocery store and when eating out. They found that mothers talked about using low-cost ingredients they were sure family members would eat and in doing so, sticking closely to planned meal rotations [32]. However, as reported by Bowen, Elliot and Brenton, pleasing family members with HPM can also be a burden to low-income women [29]. As a result of that perceived burden, mothers relied on “go-to” recipes customized to the preferences of family members, especially their children; however, in contrast to our FG participants, these mothers avoided trying new recipes [29,33]. Importantly, the “go-to” recipes our participants described were nutritionally limited and not aligned with their stated intentions of adopting healthier eating habits. This disconnect between healthy eating intention and actual intake has been reported by other researchers, though not in regard to recipe selection specifically [34]. Health professionals might assist interested mothers in adding a healthy, customizable new recipe to the family’s “go-to” recipe rotation or make a healthy adaptation of an existing favorite recipe.

Participants reported that their mothers’ and/or grandmothers’ cooking methods and styles contributed greatly to their own “go-to” recipe selections. For example, participants frequently brought up cherished childhood memories of bonding with mothers and grandmothers as they watched and helped them cook. Research indicates that childhood family food rituals are maintained into adulthood [35]. Research also indicates that fewer students learn to cook in traditional home economics classes or other schooling and adults now primarily learn cooking skills from family members and through self-teaching. This reduced exposure to formal cooking classes matters, as having cooking skills is related to healthier dietary intake in the US [36,37]. 

Research about a generational flow of recipes is emerging. Wolfson et al. examined US adults (parenthood status unreported) using FGs (*n* = 50) and a web survey (*n* = 1112; 32% low-income) to determine where participants learned to cook [38]. Results showed that a majority of participants learned to cook from their parents (66%), especially their mothers and women more so than men (72% versus 61%, respectively) [38]. Within that sample, Wolfson et al. also found that cooking knowledge and recipes passed down by family members were largely reserved for special occasions rather than daily meals [38]. Although our own FG participants did describe generational special occasion recipes like “grandma’s old-fashioned rice pudding,” they were more likely to mention nutritionally limited generational “go-to” recipes, like stroganoff, biscuits and gravy and “mom’s macaroni and cheese.” Our study provides evidence that, among Oregon families, a flow of recipes exists through at least three generations. 

Our participants talked about engaging their children in recipe selection, meal planning and cooking as a way to manage ingredient aversions and increase recipe acceptance. There is growing evidence that involvement in food preparation and development of cooking skills by adolescents and young adults is associated with healthier current and long-term eating habits [39,40,41]. Research also stresses the importance of raising awareness among parents of eating habits they model at home, the foods they have readily available in the home and the parent-child feeding relationships, as observational learning shapes both children’s immediate eating patterns and overall health trajectories into adulthood [42,43]. Based on these data, it is important that our SM campaign include customizable and healthy recipes that can become part of a family’s ‘go to’ recipe inventory but also encourage children to participate in the preparation. Recipes that meet these two criteria may help ensure that children are engaged in the meal planning and cooking and that they incorporate healthy eating behaviors and skills into their lives at a young age. 

The majority of our FG participants actively sought out new recipes and preferred to access them online, especially via recipe websites. Participants had four main criteria for desired characteristics of recipe websites. They wanted websites to be simple yet polished, with eye-catching appeal; to have an efficient search mechanism that makes recipes easy to find when entering a recipe name using a search engine such as Google; to engage children; and to offer an active user-rating system and/or text-based comments.

Although our recipe website research is novel, our results are similar to those found for general use of websites. First, our FG participants wanted a simple yet polished and eye-catching appearance in a recipe website. This is comparable to research showing that people form value judgments of online content in microseconds and their initial impressions—based largely on appearance—carry over into other evaluations of the website’s attributes and content [44]. This finding also agrees with research by Seckler et al., who found that study participants (*n* = 194) gave higher aesthetic ratings to bright and colorful websites with high symmetry and low complexity [45]. Second, our participants overwhelmingly used general search engines like Google to find recipes, rather than relying on direct visits to recipe websites or searches within recipe websites, even familiar websites. Similarly, Kennedy et al. found that pregnant women from diverse socioeconomic backgrounds in Ireland (*n* = 101) relied heavily on Google searches to find relevant online nutritional information [46]. Egri and Bayrakb (2014) also found that 93% of Internet traffic can be attributed to search engine use; thus, affirming the importance of search engine optimization and key word searches to bring new and return users to a website and keep them there [47]. Third, our participants placed high value and trust in evaluations of online recipes by others, including from star-rating systems and text-based reviews. Research shows that social validation through user ratings can increase the perceived credibility of online health information [48,49]. To our knowledge, our other findings specific to recipe websites have not previously been cited in the literature. These include insights that participants value the ability to refine recipe searches with key words and desire more child-centered content on parent-targeted recipe websites. 

### 4.1. Study Strengths

This research has four key strengths. First, to our knowledge this is the first study to focus on describing the characteristics low-income mothers look for in recipes, including characteristics valued by mothers when selecting recipes online. Second, we used five years (2011–2015) of FG data to examine how low-income mothers select and use recipes for HPM. Third, results from the FGs provided insights that have been used to continually refine the content and delivery of recipes through the ongoing Food Hero SM campaign. Fourth, only SNAP-enrolled participants were recruited through a strong partnership with Oregon’s DHS.

### 4.2. Limitations

These findings used subjective narratives to provide unique insights about recipe use among English-speaking, low-income mothers; thus, they cannot be generalized to all households. It is possible those who attended the FGs are a sub-set of the population who rely more heavily on HPM compared to the general SNAP population. Findings related to a distinct subset, however, may still have useful implications for a broader audience when viewed through a social cognitive lens of sharing and modeling HPM strategies and observational learning through successful outliers [25]. FG dynamics are also subject to social desirability bias; it is therefore possible that participants did not mention some of their more socially undesirable habits related to choosing and preparing meals or other salient aspects of their home food environments in general [50]. Future researchers may want to consider similar research questions using face-to-face interviews to gain further insights.

### 4.3. Implications for Research and Practice

Despite routine promotion of recipes within nutrition and public health programs as a way to encourage HPM and healthy eating, there is no consensus regarding best practices for types of healthy recipes to offer, nor how or to whom to deliver them. Our research identified criteria used by low-income Oregon SNAP eligible mothers who generally cook HPM often and enjoy cooking, when searching for and choosing new and “go to” recipes. This study underscores that these mothers customize recipes to fit within their family’s unique constellation of preferences and circumstances and thus are not looking for one-size-fits-all prescriptive recipes. Rather, to Oregon SNAP eligible mothers who regularly cook HPM, health educators should consider promoting low-cost, easily customizable, healthy recipes that use common ingredients and can be delivered on interactive platforms to encourage self-efficacy and child involvement with planning/cooking. Strategies should recognize that children and adults are key influencers of current and future family food environments. Our findings also point to the potential need for search engine optimization of healthy online recipes to make them easier and more convenient to find. 

Since the research described in this paper was conducted, results have been used to develop two adult intercept surveys that aim to empirically test related research questions with a larger sample size. Future research in this area should focus on input from diverse population segments about desired recipe traits, readiness to try new healthy recipes and adapt existing recipes to be more healthful and best ways to access new recipes and recipe healthy adaptation tips. Additionally, nutrition educators can encourage online promotion of healthy recipes by using bright and polished photographs, facilitating high name recognition in Google searches and incorporating interactive engagement opportunities like user comments and ratings. More research, however, is needed on what combination of qualities and delivery mechanisms is ideal. Gathering a target audience’s input about desired recipe traits and how best to deliver recipes is an important formative step of obesity prevention projects that include recipes as a tool to promote healthy eating habits.

## Figures and Tables

**Table 1:** Demographics of focus group (FG) participants (*N* = 55).

FG	Date	Start Time (90-min Sessions)	Participants (*n*)	Mean Age (y)	Households with 3–4 People (%)	Households with 1–2 Children (%)
1	02/11/11	11:00 AM	6	36.7	65	76
2	09/17/13	11:00 AM	9	36.8	53	53
3	09/17/13	1:00 PM	6			
4	09/18/13	11:00 AM	8	39.3	36	43
5	09/18/13	1:00 PM	6			
6	09/29/14	10:00 AM	4	38.3*	54	71
7	09/29/14	12:30 PM	4			
8	12/17/15	5:30 PM	5	35.3*	58	83
9	12/17/15	7:30 PM	7			
Totals			*N* = 55	~ STD = 8.75 y	53%	65%

* Estimated mean is given when participant data were reported as age ranges.

## Supplementary Materials

The following are available online at http://www.mdpi.com/2072-6643/11/2/339/s1, Table S1. Structural model of current attitudes toward healthy eating, recipe use and selection, use of recipe websites and related factors and corresponding focus group questions administered to low income mothers living in Tri-county Portland area, Oregon, USA, 2011–2015.
