# Supplementary material for: How Low-Income Mothers Select and Adapt Recipes and Implications for Promoting Healthy Recipes Online
Source: Nutrients. 2019 Feb 5;11(2):339. doi: 10.3390/nu11020339 (PMC6412388; doi:10.3390/nu11020339)
Supplement: Supplementary file 1 [file nutrients-11-00339-s001.pdf]

Supplement.

Table S1. Conceptual categories of current attitudes toward healthy eating, recipe use and selection, use of recipe websites and related factors, and corresponding focus group questions administered to low income mothers living in Tri-county Portland area, Oregon, USA, 2011–2015.

**Conceptual Categories**	**Focus Group Questions**
Home prepared meals self-efficacy, frequency, and food preparation attitudes	• How do you decide what to cook and serve your family for each meal?<br>• How often do you cook meals at home?<br>• What is your favorite meal to cook? What sorts of things do you like to cook for your family?<br>• What do you/your kids like to eat?<br>• Are there some things that you're comfortable with your kids doing in the kitchen, and other things you aren't?<br>• Which ways do your kids like to help you cook?
Healthy eating definitions and perceived barriers to healthy eating	• What does healthy eating mean to you?<br>• Is healthy eating the same for you as it is for your kids?<br>• Do your kids eat well? Do they eat the things you would like them to eat?<br>• Are there any foods you're not sure if they're healthy?<br>• What makes a recipe healthy?<br>• What makes it easier or more difficult for you to cook healthy? Does anyone have things they like to do for cooking, but also struggle with?
Recipe use and selection	• Do you use recipes when cooking?<br>• Where do you find the recipes you use?<br>• Tell me about the types of recipes you get off the Internet – be specific.<br>• Do you include your kids in the process when you pick recipes?<br>• When you are searching for recipes, how do you find that information? What recipe websites do you go on?
Current use of recipe websites and desired traits in style and content	• What websites do you go to for recipes or information about food or cooking for your family?<br>• What are some important pieces that should be on a website about recipes?<br>• Are there websites that really capture your attention? What keeps you focused on them?<br>• What makes a recipe or food website kid-friendly?<br>• Suppose you wanted to design a website to get people like you to find recipes and meal ideas, with the goal of encouraging families to eat healthy, including more fruits and vegetables. What would the website include?

Food Hero campaign elements, messaging, and recipe delivery	• What would you expect to find in a newsletter called *Food Hero Monthly*?<br>• What would make Foodhero.org more attractive than other recipe websites?<br>• What are your general impressions of Foodhero.org? Food Hero recipe books? *Food Hero Monthly*?<br>• Do you have feedback about the look, the feel, the pictures on the *Food Hero Monthly*?
